# The hidden community architecture of human brain networks

**DOI:** 10.1038/s41598-022-07570-0

**Published:** 2022-03-03

**Authors:** Byeongwook Lee, Uiryong Kang, Hongjun Chang, Kwang-Hyun Cho

**Affiliations:** Laboratory for Systems Biology and Bio-inspired Engineering, Department of Bio and Brain Engineering, Korea Advanced Institute of Science and Technology (KAIST), Daejeon, 34141 Republic of Korea

**Keywords:** Computational neuroscience, Neural circuits, Systems analysis, Computational neuroscience, Network topology

## Abstract

The organizational principles of the community architecture of human brain networks are still mostly unknown. Here, we found that brain networks have a moderate degree of community segregation but are specifically organized to achieve high community overlap while maintaining their segregated community structures. These properties are distinct from other real-world complex networks. Additionally, we found that human subjects with a higher degree of community overlap in their brain networks show greater dynamic reconfiguration and cognitive flexibility.

## Introduction

**Figure 1:**
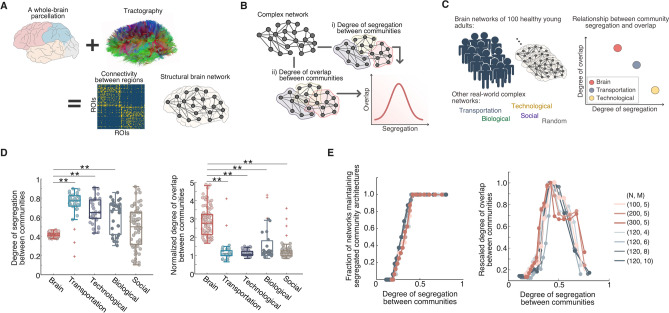
Comparison of the community architecture of brain networks and that of other real-world complex networks. (**A**) Using the structural and diffusion magnetic resonance imaging (MRI) data obtained from the Human Connectome Project database, we performed whole-brain parcellation and diffusion tractography to reconstruct structural brain networks of 100 healthy young adults. (**B**) A hidden community architecture of a complex network is characterized in a two-dimensional space by the degree of community segregation and the degree of community overlap on each axis. (**C**) By identifying the community architecture of the structural brain networks and comparing it with that of other real-world complex networks, we investigated unique characteristics of the community architecture of brain networks that may support complex brain functions. (**D**) Comparison between brain networks and other real-world complex networks. Compared to other real-world complex networks, the brain networks showed significantly lower segregation scores (left) and significantly higher overlap scores (right). Each overlap score was normalized by the average overlap score of 10 corresponding random null networks. **p < 0.001, two-tailed *t*-test. (**E**) A general relationship between the degree of community segregation and the degree of community overlap was examined using synthetic modular networks. The colors of the lines represent the results from the synthetic modular networks with different network sizes (N) and different number of modules (M). These analyses revealed that the degree of overlap between communities is maximized at a moderate degree of community segregation. Each overlap score was rescaled over the maximum value in the corresponding modular configuration (N, M).

Cognitive function emerges from elaborate interactions among groups of densely inter-connected brain regions^1,2^. These interactions constitute the complex community architecture of human brain networks^1,2^. In contrast to the conventional notion that communities must be sufficiently segregated to promote functional specialization in brain networks^2^, studies show that the communities overlap, allowing a brain region to participate in multiple functional tasks^3,4^. Understanding how the brain’s structural connectivity constitutes such a segregated but also overlapping community architecture will provide insight into how the brain performs distinct yet diverse functions. Here, we examined the degree of segregation and overlap between communities of brain networks and other real-world complex networks. We investigated how these two features interdependently constitute a community architecture of a complex network. In particular, we reconstructed the structural brain networks of 100 healthy young adults using the structural and diffusion magnetic resonance imaging (MRI) data obtained from the Human Connectome Project database (Fig. 1A, see “Methods” section for details). Using the reconstructed brain networks, we investigated whether the brain networks exhibit a unique community architecture compared to 157 other real-world complex networks (Fig. 1B and 1C). Our analysis revealed that human brain networks are distinguished from other real-world complex networks by having a high level of overlap between communities while ensuring a moderate level of community segregation. We then explored whether such a characteristic of the brain network architecture supports complex dynamic interactions between brain regions and cognitive performance, which indicated that such an organizational principle supports rich functional repertoires of brain dynamics and high cognitive flexibility.

## Results

### Comparison of the community architecture of brain networks and that of other real-world complex networks

To characterize the community architecture of human brain networks and compare it with that of other real-world complex networks, let us consider a two-dimensional space with community segregation and community overlap on each axis. We hypothesized that the degree of community overlap depends on how precisely the network can be dissociated into distinct communities. We quantified two distinct properties of community architecture with the following metrics: (1) the segregation score, which quantifies the degree of segregation between communities, and (2) the overlap score, which quantifies the degree of overlap between communities. To measure the segregation score, we calculated the modularity index of each network. The modularity index quantifies how well a network is segregated into non-overlapping communities^5^. To establish a baseline for the brain networks, we used “null” networks, which represent degree-preserved randomized networks of the corresponding brain networks. Although the brain networks had significantly higher segregation scores compared to their null networks, the brain networks had significantly lower segregation scores compared to other real-world networks (Fig. 1D left, see Table S1, Fig. S1 and S2). Next, we computed the overlap score of each network using the overlapping community detection algorithm^6^ (see “Methods” section for details) by measuring how many different communities each network node belongs to on average. This analysis revealed that the brain networks had significantly higher overlap scores than their null networks as well as than other real-world networks (Fig. 1D right, see Table S1 and Fig. S2). These results indicated that the brain networks exhibit a distinctive community architecture compared to other real-world complex networks.

To identify a relationship between the degree of community segregation and the degree of community overlap, we generated synthetic modular networks with different segregation scores and examined their overlap scores. Our analysis showed that the number of overlapping communities becomes equal to or larger than the number of non-overlapping communities at a moderate level of the segregation score (Fig. 1E left, see “Methods” section for details). Further analysis revealed that overlap scores exhibit a bell-shaped change as segregation scores increased, showing that the degree of community overlap in a network is maximized specifically at the moderate range of community segregation scores (Fig. 1E right). Interestingly, this specific range corresponded to the community segregation scores observed in the brain networks. Taken together, these results suggested that interconnectivity of the brain networks might have evolved to enable a highly overlapping community architecture while maintaining their segregated community structures.

### Optimal community architecture in brain networks

**Figure 2:**
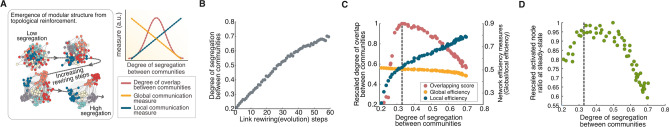
Optimal community architecture in brain networks. (**A**) Schematic illustration of an approach to investigate the relationship between the degree of community segregation, the degree of community overlap, and network measures that characterize communication efficiency. (**B**) Evolution of the degree of segregation between communities in the TR model as a function of the number of rewiring steps. (**C**) Global efficiency and local efficiency are both optimized at the point where the degree of overlap between communities is maximized. (**D**) Activated node ratio at steady state and the degree of overlap between communities exhibit similar bell-shaped changes as community segregation score increases. In (**B**), (**C**), and (**D**), each dot represents the average result of 50 evolving networks located at the same evolution step. In (**C**) and (**D**), the overlap scores were rescaled over the maximum value, and the black dashed line indicates the point of maximum overlap score. The proportion of seed nodes in this figure was set to 32%.

What would be the benefit of having a highly overlapping community architecture? We explored this question by examining how network communication efficiency depended on the community architecture of a network. To investigate multiple model networks with different community architectures, we employed the topological reinforcement (TR) model that mimics the emergence of community architecture in brain networks during a neurodevelopmental process (Fig. 2A)^7^. By promoting connections between nodes that share more common neighbors than others, the TR model mimics the Hebbian rule in its rewiring process and produces modularized (that is segregated) networks^7^. The TR model can evolve initial networks with a random structure into networks with greater segregation (Fig. 2B). With each of the model networks generated during the rewiring process, we calculated the following communication efficiency measures: (1) global efficiency, (2) local efficiency, and (3) the ratio of activated nodes at a steady state. Whereas the first two measures quantify global and local communication efficiencies on the basis of routing-based strategies, the last measure quantifies communication efficiency on the basis of diffusion-based strategies^8^. Both routing-based and diffusion-based communication are employed in human brain networks^8^. Based on previous findings suggesting that the brain has an ability to balance segregation and integration of communities for efficient information processing^1^, we hypothesized that there are functional benefits in brain networks having a high overlap score. Our analysis indicated that routing-based global and local communication are optimized at degree of segregation where the overlap score is maximal (Fig. 2C). This suggested that the community architecture of these networks simultaneously optimizes both forms of communication in the brain. Furthermore, the ratio of activated nodes at a steady state exhibited a similar pattern as that of the community overlap score as community segregation increases (Fig. 2D), suggesting that the community architecture of brain networks is designed to minimize the cost of activating the entire system. Taken together, these results implied that the highly overlapping community architecture of brain networks results in functional advantages with respect to network communication.

### The relationship between the community architecture and the dynamics or cognitive flexibility of the human brain

**Figure 3:**
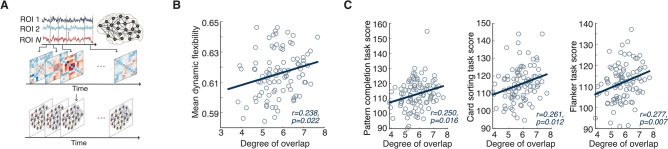
The relationship between the community architecture and the dynamics and cognitive flexibility of the human brain. (**A**) To investigate a structure-dynamic relationship of human brain networks, we calculated mean dynamic flexibility of functional brain networks. Mean dynamic flexibility is defined as the mean rate of transitions of each brain region between different network communities. (**B**) The degree of overlap between communities in the structural brain networks is positively correlated with the mean dynamic flexibility. (**C**) The degree of overlap between communities in the structural brain networks is positively correlated with the behavioral measures that are associated with cognitive flexibility.

We examined the association between the community architecture of brain networks and brain dynamics. Brain dynamics are characterized by dynamic reconfiguration of functional interactions between distributed brain regions. A higher dynamic reconfiguration rate is associated with better cognitive performances^9^. However, how the structural brain network supports such a dynamic property is still poorly understood. We hypothesized that individuals with a higher degree of community overlap in their brain networks would have a greater dynamic reconfiguration rate. We tested this hypothesis using the time-varying functional connectivity estimated from resting-state functional MRI (rfMRI) data of the same 100 subjects used to evaluate the properties of structural brain networks. The time-resolved functional connectivity data were used to compute a mean dynamic flexibility^9^, a measure used to examine an averaged dynamic reconfiguration rate of a brain network (Fig. 3A). Intriguingly, the overlap score is positively correlated with the mean dynamic flexibility of the networks (*r* = 0.238, *p* = 0.022, Fig. 3B and S3), indicating that individuals with a higher degree of community overlap in their brain networks have higher flexibility in functional interactions between distributed brain regions. In contrast, the community segregation score and the mean dynamic flexibility are negatively correlated (Fig. S4). Taken together, these results suggested that the overlapping community architecture of structural brain networks represents a hidden structural principle for highly dynamic reconfiguration of inter-regional interactions.

Next, we investigated whether an inter-individual difference in the degree of community overlap is associated with behavioral task performance. In particular, we hypothesized that the subset of behavioral tasks important for cognitive flexibility would have a significant relationship with the degree of community overlap, because such overlap would support flexible transitions between different cognitive states. We examined 3 cognitive tasks: (1) the pattern completion task that indicates processing speed, (2) the card sorting task that indicates cognitive flexibility, (3) the flanker task that indicates cognitive inhibition. Correlation analyses revealed that the overlap score is positively correlated with the scores of these cognitive tasks (Pearson’s correlation between the overlap score and the pattern completion task: *r* = 0.250, *p* = 0.016; Pearson’s correlation between the overlap score and the card sorting task: *r* = 0.261, *p* = 0.012; Pearson’s correlation between the overlap score and the flanker task: *r* = 0.277, *p* = 0.007) (Fig. 3C). In contrast, no significant relationship was found between the segregation score and the scores of the pattern completion task and the flanker task (Fig. S5). Additional analysis on the group-averaged brain network (the consensus brain network) shows that brain regions with a high degree of overlap, including superior frontal gyrus, precuneus, putamen and thalamus, are located either in fronto-parietal or subcortical regions and are closely associated with cognitive flexibility, a core dimension of executive functions (Fig. S6)^10–13^. Taken together, these results suggested that higher overlap between communities in structural brain networks supports better cognitive performance.

## Discussion

Community architecture is a core design principle of human brain networks. Typically, the community architecture of brain networks has been described by assigning each brain region to a single community that supports a single specialized function^1,2^. However, many brain regions could engage in multiple brain functions, suggesting that a non-overlapping community architecture might be insufficient to explain the rich repertoires of brain dynamics^14,15^. In this study, we investigated an organizational principle of community architecture in human structural brain networks and its association with brain dynamics and cognitive functions. The principle is based on a unified framework that accounts for both community segregation and overlap. We found that the unique community architecture of human brain networks, characterized by a moderate degree of community segregation and high overlap between communities while maintaining its segregated community structure, could provide a basis for the diverse repertoires of brain dynamics and high cognitive flexibility. In this regard, our study contributes to bridge the gaps among brain structure, its dynamics, and cognitive functions by uncovering the hidden community architecture of human brain networks.

Although network binarization has an important advantage of being able to use a variety of topology metrics and demonstrate robustness against measurement noise^16^, recent studies have begun to consider the directionality and the weight of connections between brain regions^17–21^. For example, de Lange et al. performed weighted and binary network analysis on the macaque connectome, and observed a small-world, modular and rich-club organization in both analyses^21^. Choosing a suitable attribute for connection weights (i.e. strengths, distances) and a proper imaging modality for quantifying inter-regional connectivity has recently received the attention of many researchers. In this regard, employing weighted brain networks in investigating the community architecture could provide a deeper understanding of complex brain functions.

A coherent line of research has investigated the relationships among brain structure, function and cognition, based on the idea that structural characteristics of the brain provide the basis of complex brain functions^22–25^. As a core component of executive functions, cognitive flexibility often requires coordinated actions of multiple brain regions^13^. In this respect, the overlapping community architecture of brain networks, which allows each brain region to be overlapped in multiple communities, could provide a more versatile physiological basis for cognition. In this study, we demonstrated that individuals of healthy subjects that have a lower degree of segregation and a higher degree of overlap could perform better in high-order cognitive tasks (Fig. 3C and S5). Further investigation of the relationship between community structure and more diverse levels of cognitive tasks could advance our understanding of the functional advantages of the overlapping community architecture in brain networks.

In the analysis of the community architecture of the consensus brain network, we found that highly overlapped brain regions are the areas closely related to the cognitive flexibility^10–13^. However, in the process of brain development or neurodegenerative disease, the structural connectivity of a brain, especially for these key regions, undergoes significant changes. For example, the volumes of putamen and thalamus are significantly reduced in Alzheimer's disease patients, which eventually leads to impaired global cognitive performance^10^. In this context, it is expected that further studies characterizing changes in the community architecture of structural brain networks during developmental and degenerative processes can advance our current understanding of complex neurodevelopmental and neurodegenerative disorders.

## Methods

### Brain network reconstruction

Structural brain networks were reconstructed from the structural and diffusion magnetic resonance imaging (MRI) data provided by the Human Connectome Project (HCP) database^26^. Individual nodes and links in the networks were chosen to represent brain regions and anatomical connections between them. The HCP scanning protocol was performed in compliance with the approval of the local institutional review board at Washington University in St. Louis. From the database, the preprocessed structural and diffusion MRI data of 100 unrelated subjects from HCP 1200 Subject Release was used to reconstruct the structural brain networks of 100 healthy young adults. The full details of the preprocessing pipeline have been described in the previous study^27^. Reconstruction of the structural brain networks was carried out through the following procedure by using the MRtrix3 package^28^: (1) Generate a tissue-segmented image from structural MRI data for Anatomically-Constrained Tractography (ACT)^29^; (2) match the parcellated brain image to the Destrieux atlas^30^, which consists of 164 cortical and subcortical regions; (3) calculate the fiber orientation density (FOD) with diffusion MRI data by multi-shell, multi-tissue constrained spherical deconvolution (MSMT-CSD)^31^; (4) trace white-matter neural fibers connecting each pair of brain regions by using probabilistic tractography algorithm^32^; (5) construct the adjacency matrices for each structural brain network by enumerating the number of neural fibers between brain regions. The overall streamline count number was set to 10 million, which is the default value of the MRtrix3.

We binarized each brain network to the presence or absence of inter-regional connectivity, to compare brain networks with various types of binarized real-world complex networks and to solely focus on investigating the contribution of connectivity pattern in determining community architecture. If connectivity exists between region *i* and *j*, the value of the corresponding network element is set to 1; otherwise, the value is set to 0. We set each brain network to have a link density of 10%, and further reconstructed a group-averaged brain network (the consensus brain network) by selecting all connections that are present in at least 75% of the 100 subjects. Through this process, each of the reconstructed brain network and the group-averaged brain network are realized in the form of an undirected and unweighted network. The community architecture of brain networks was also determined at different link densities (Fig. S2).

### Network collection

For comparison with the structural brain networks, we collected 157 real-world complex networks from 4 disparate fields, ranging from transportation networks (31 networks), technological networks (36 networks), biological networks (30 networks), and social networks (60 networks). These networks were collected from the Stuart et al*.*^33^, and were also used in the form of an undirected and unweighted network for further analysis. Details of each network are summarized in Table S1.

We generated 100 randomized and 100 latticized (i.e. grid-structured) null networks derived from the group-averaged brain network of 100 subjects, which we described above. Each type of null network was generated using the functions *randmio_und_connected* or *latmio_und_connected* in the Brain Connectivity Toolbox^34^. These functions randomly permute the edges of a network or latticizes a network while preserving the number of nodes, links, and its degree distribution.

We also prepared synthetic modular networks with different community segregation scores. Model networks with equally-sized modules were created by manipulating the probability of within- and between-community edge placement (Code is available at http://github.com/macshine/integration/guimera_model.m)^35^. The probability of within-community edge placement was varied between 0.7 and 1 with 0.02 intervals, and the probability of between-community edge placement was varied between 0.01 and 0.35 with 0.02 intervals. To investigate a general relationship between the degree of community segregation and overlap, the values of the two probabilities were obtained across the parameter space for generating each synthetic modular network. We used 7 different modular configurations in this study; (the number of nodes N, the number of modules M) = (100, 5), (200, 5), (300, 5), (120, 4), (120, 6), (120, 8), and (120, 10).

### Community architecture in complex networks

To characterize the community architecture of human brain networks and compare it with that of other real-world complex networks, we quantified two distinct properties of community architecture with the following metrics: (1) the segregation score, which quantifies the degree of segregation between communities, and (2) the overlap score, which quantifies the degree of overlap between communities. To measure the segregation score, we partitioned a complex network into non-overlapping communities and computed the modularity index using the Louvain community detection algorithm (gamma = 1)^5^, as implemented in the Brain Connectivity Toolbox^34^. The resultant modularity index was utilized as a segregation score of the network. To measure the overlap score, we partitioned a complex network into overlapping communities using the link community algorithm^6^, as implemented in the Brain Connectivity Toolbox^34^. Then, we quantified the overlap score by calculating the average number of different communities to which each node belongs. In the process of calculating the overlap score, we ensured that each node in a network belongs to at least one community. We also examined the number of overlapping communities of each network to check whether it maintains its segregated community structure. If the number of overlapping communities of a network is equal to or higher than the number of predefined non-overlapping modules, we determined that the network maintains its segregated community structure. In addition, when comparing network groups with different attributes, such as brain networks and real-world complex networks, the overlap score of each network has been normalized by the average overlap score of its 10 random null networks for group-wise comparisons.

### Topological reinforcement model

To analyze the functional benefits of human brain network community architecture, we employed the topological reinforcement (TR) model, which mimics activity-dependent adaptation and synaptic plasticity during a neurodevelopmental process. By promoting connections between nodes that share more common neighbors than others, this model reliably evolves initial networks with a random structure into more modularized networks and supports the role of TR as a contributor to the emergence of modular brain networks. A more detailed description of the TR model is given in the previous study^7^.

We prepared 50 Erdős–Rényi (ER) random networks without self-connections of size N = 164 nodes and average node degree $\lambda$ = 16 (equivalent to a density of 10%). At each rewiring step, we randomly selected N/2 nodes that are neither disconnected nor fully connected to all other nodes. Then we added simultaneously one link on each selected node with a non-neighbor that has the highest topological overlap score. The topological overlap score represents the neighborhood similarity of a pair of nodes by counting the number of their common neighbors^7^. After inserting links, the same number of links were randomly eliminated to maintain the original link density. We set the number of evolution steps to 60 so that sufficient link rewiring can take place for modularity emergence as long as the network is connected. In each rewiring step, we collected resultant networks to analyze the functional benefits of human brain network community architecture.

### Graph theoretical analysis

All graph-theoretical analyses were performed using the Brain Connectivity Toolbox^34^ (http://sites.google.com/site/bctnet/).

### Linear threshold model

We employed the linear threshold model to compare communication efficiency in view of network diffusion. In this model, every node of a network has a common threshold parameter within [0, 1] and a certain percentage of nodes are selected as ‘seed nodes’ for initial stimuli. An activated node can have 1/node_degree of influence on each of its neighbors, and the influence a given node receives is calculated by summing the influences from its activated neighbors. If the influence received by a given node exceeds a predefined threshold parameter, it becomes an activated node that can influence its neighbors. This chain reaction is finished until there is no more changes in the number of activated nodes in a network, which is referred to as a steady state (code is available at https://github.com/hhchen1105/networkx_addon/information_propagation/linear_threshold.py)^36^.

We varied the proportion of seed nodes from 5 to 50% and set a threshold parameter to 0.5. We randomly selected seed nodes and then simulated a diffusion process until the number of activated nodes in a network no longer changes. Communication efficiency in view of network diffusion was quantified by the ratio of activated nodes at a steady state.

### Dynamic flexibility of functional brain networks

To examine the dynamic flexibility of functional brain networks of the 100 subjects, we used each subject’s resting-state functional MRI (rfMRI) time-series data (sampling rate = 0.72 s.) that are publicly released through the central HCP Connectome DB database. First, the time-varying inter-regional functional connectivity was estimated by correlation-based sliding-window analysis using the time-series data with a different number of independent component analysis (ICA) dimensionalities (N = 50 or 100). This results in 50- or 100-node time-series of 4,800 time points for each subject. We used a window length of 30 s. and each window was shifted 0.72 s. across the whole scan, resulting in a total of 4759-time windows (W). We generated a 3D matrix of correlation coefficients of size N × N × W, composed of 2D N × N matrices for every time window W. Using the dynamic community detection algorithm^5^, we identified network modules in each time window and tracked their evolution over time. By estimating the probability that a brain region changes its allegiance to modules between any two consecutive time windows, we computed the dynamic flexibility of each brain region. To investigate a structure–function relationship, we correlated the mean dynamic flexibility of the functional brain networks and the overlap score of the 100 structural brain networks (Fig. 3B and S3).

### Behavioral measures of cognitive flexibility

We used three behavioral measures of cognitive tasks that enabled the examination of the three aspects of cognitive flexibility: cognitive inhibition, executive function, and processing speed. These cognitive tasks include (1) the pattern completion task (processing speed), (2) the card sorting task (executive function), and (3) the flanker task (cognitive inhibition)^37^. The behavioral measures of each task are recorded for each HCP participant. These measures are part of the HCP NIH toolbox, which is a multidimensional set of behavioral measures evaluating diverse aspects of cognitive functions^26,37^. To investigate a relationship between the community architecture and cognitive flexibility, we correlated the performance score and the overlap score of the 100 structural brain networks in each cognitive task.

### Statistical analysis

We performed two-tailed *t*-tests to determine whether the characteristics of the given network groups shows a significant difference in the perspective of community architecture.

## Supplementary Information

Supplementary Information 1.

Supplementary Information 2.
