# Supplementary material for: The hidden community architecture of human brain networks
Source: Sci Rep. 2022 Mar 3;12:3540. doi: 10.1038/s41598-022-07570-0 (PMC8894465; doi:10.1038/s41598-022-07570-0)

The hidden community architecture of human brain networks

Supplementary Information

*Byeongwook Lee¶, Uiryong Kang¶, Hongjun Chang, Kwang-Hyun Cho**

Laboratory for Systems Biology and Bio-inspired Engineering, Department of Bio and Brain Engineering, Korea Advanced Institute of Science and Technology (KAIST), Daejeon 34141, Republic of Korea

¶These authors contributed equally to this work.

*Corresponding author. E-mail: ckh@kaist.ac.kr, Phone: +82-42-350-4325, Fax: +82-42-350-4310, Web: http://sbie.kaist.ac.kr/

Figure S1. Comparison of the degree of segregation between brain networks and other real-world complex networks. (A) A distribution showing network sizes of 157 real-world complex networks used in this study. **(B)** Correlation between degree of segregation and log-transformed network size of 157 real-world complex networks. Each circle represents a real-world complex network analyzed in this study. **(C)** Correlation between degree of segregation and log-transformed network size of 51 real-world complex networks that are similar to the size of the brain networks. **(D)** Degree of segregation in brain networks and other real-world complex networks with between 100 and 300 nodes. ***p*<0.001, two-tailed *t*-test.

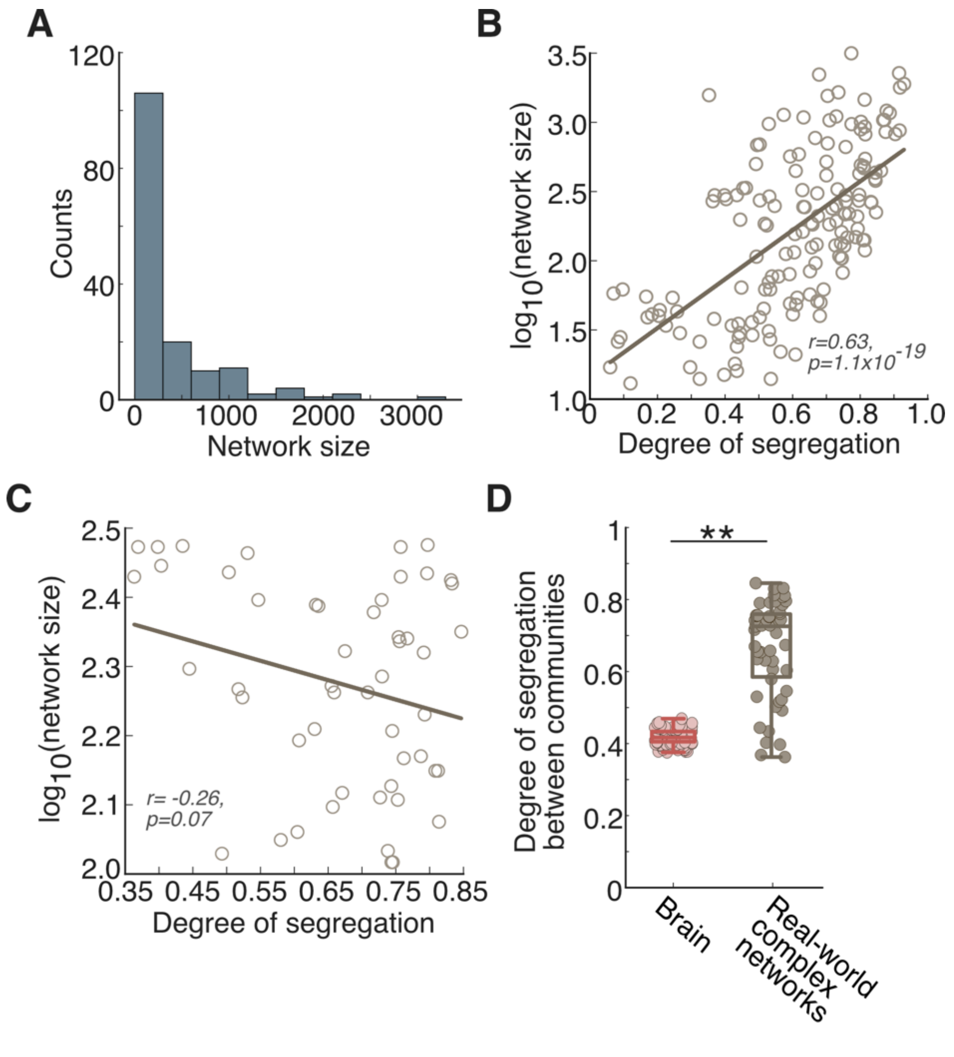

Figure S2. Comparison of the community architecture of brain networks and that of their null networks with different link densities. (A) Brain networks showed a significantly higher degree of segregation between communities than that of their latticized (latt.) or randomized (rand.) null networks with different link densities. **p<0.001, two-tailed t-test. (**B)** Brain networks showed a significantly higher degree of overlap between communities than that of their latticized or randomized null networks with different link densities. **p<0.001, two-tailed t-test. Each group consists of 100 networks with link densities set at 10%, 15%, and 20%.

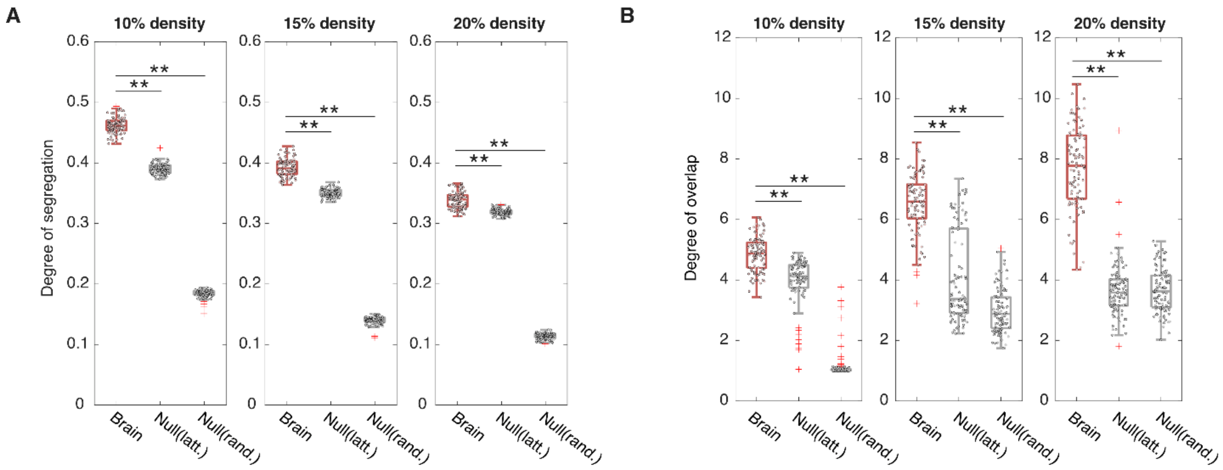

Figure S3. The relationship between the degree of overlap and the mean dynamic flexibility of 100 healthy young adults. The mean dynamic flexibility of each subject was calculated from time-varying inter-regional functional connectivity, estimated from time-series data with a different number of independent component analysis (ICA) dimensionalities (*N*=50, 100). The relationship between the degree of overlap and the mean dynamic flexibility was replicated in all the ICA dimensionalities (*N*=50, 100) tested in this study.

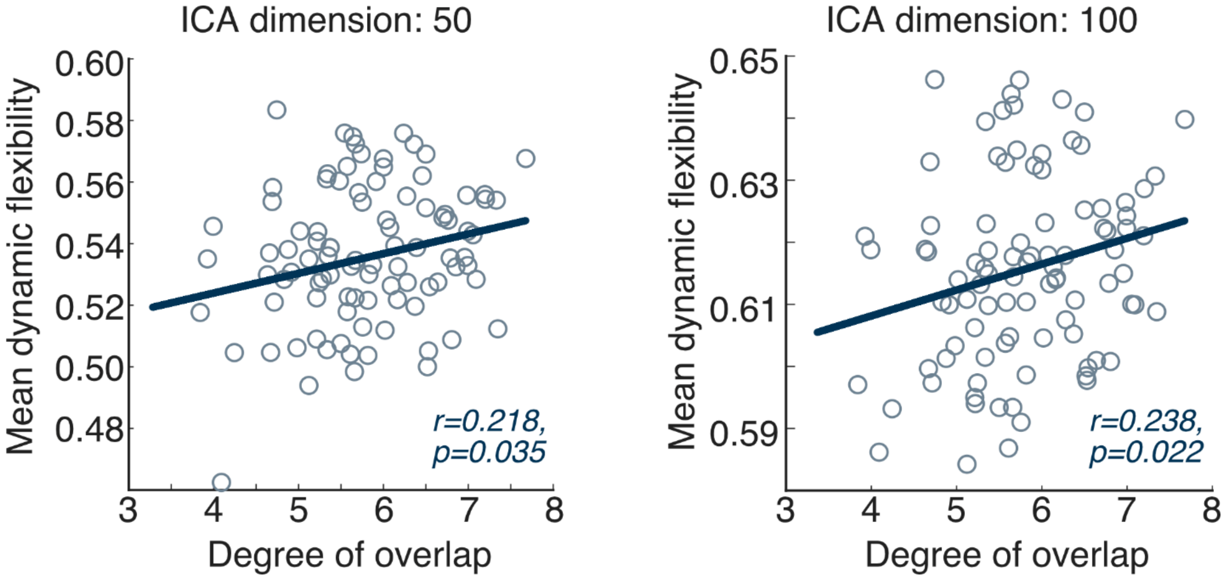

Figure S4. The relationship between the degree of segregation and the mean dynamic flexibility of 100 healthy young adults. The mean dynamic flexibility of each subject was calculated from time-varying inter-regional functional connectivity, estimated from time-series data with a different number of independent component analysis (ICA) dimensionalities (*N*=50, 100). The relationship between the degree of segregation and the mean dynamic flexibility was replicated in all the ICA dimensionalities (*N*=50, 100) tested in this study.

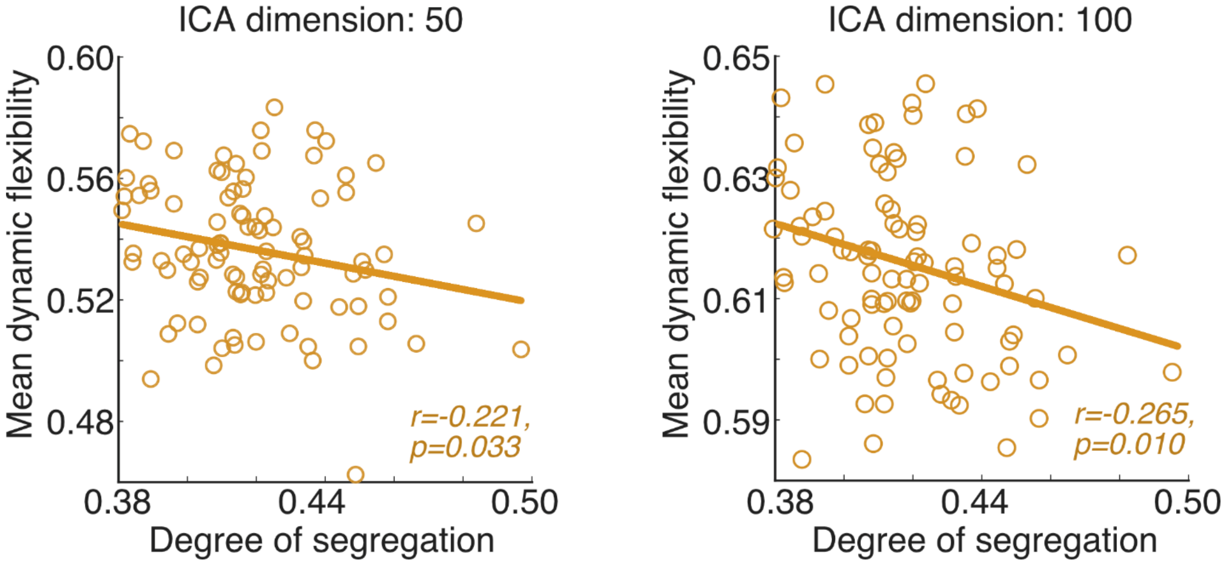

Figure S5. The relationship between measures associated with cognitive flexibility and the degree of segregation between communities.

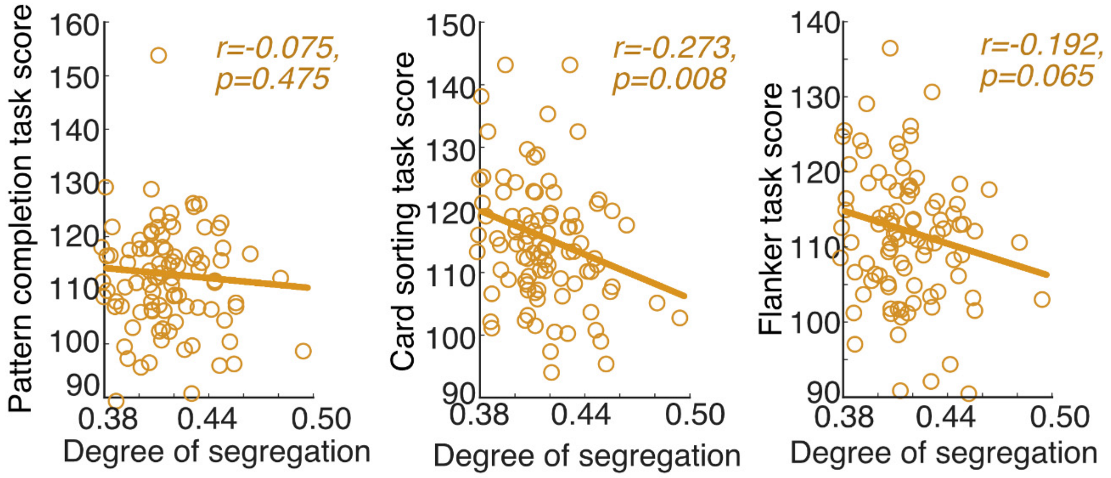

Figure S6. The community architecture of the consensus brain network. (A) Non-overlapping community architecture of the consensus brain network with the standard resolution parameter of gamma equal to 1. Each of the five modules is marked in a different color. **(B)** The number of community overlaps for each node in the same consensus brain network. **(C)** The distribution of the number of community overlaps for each node. **(D)** Top 10 brain regions with the highest number of community overlaps.

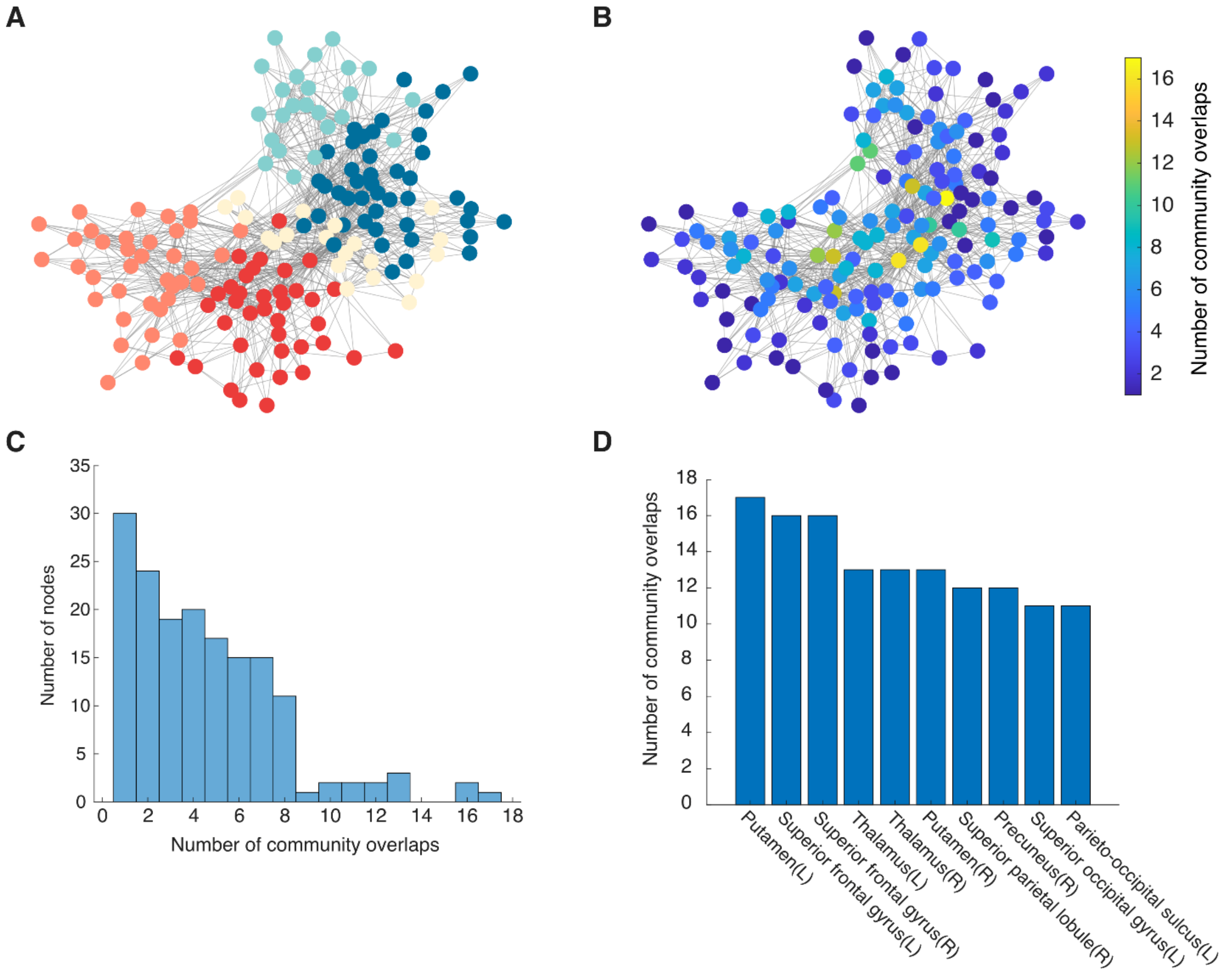

Supplement: Supplementary file 1 — Supplementary Information 1. [file 41598_2022_7570_MOESM1_ESM.pdf]
